# Estrogenic or antiestrogenic therapies for multiple myeloma?

**DOI:** 10.1186/1476-4598-6-59

**Published:** 2007-09-24

**Authors:** Brigitte Sola, Jack-Michel Renoir

**Affiliations:** 1 Biologie moléculaire et cellulaire de la signalisation, IFR 146, Université de Caen, Caen, France; 2 Pharmacologie cellulaire et moléculaire des anticancéreux, CNRS UMR 8612, Châtenay-Malabry, France; 3 Université de Paris-Sud, IFR 141, Orsay, France

## Abstract

Multiple myeloma (MM) is a common hematological malignancy which remains incurable due to both intrinsic and acquired resistance to conventional or more novel drugs. Estrogenic and antiestrogenic compounds are very promising drugs for the treatment of MM. Indeed, they inhibit cell proliferation *in vitro*. They block cell cycle and/or induce apoptosis even in drug-resistant MM cells but not normal B cells. They interfere with survival pathways often deregulated in myelomas. They co-operate with conventional drugs to enhance apoptosis or to overcome resistance. *In vivo*, they act also on tumoral angiogenesis in xenograft models. As a whole, they possess all the criteria which render them attractive for a new therapeutic strategy. Importantly, they are well-tolerated at the doses tested *in vitro *or *in vivo*, encouraging the rapid onset of critical trials.

## Review

### Introduction

Multiple myeloma (MM) is still an incurable malignancy characterized by the accumulation of tumoral plasma cells in the bone marrow. This accumulation of myeloma cells results in the overproduction of monoclonal immunoglobulins and bone destruction, two clinical features of the disease [1]. Malignant plasma cells and bone marrow stromal cells establish multiple interactions through adhesion molecules and growth factors which both activate complex signaling pathways that sustain survival of malignant cells, mediate tumor progression and drug resistance [2]. Thus, to be effective in MM, therapeutic agents must target both myeloma cells and bone marrow environment. 2-methoxyestradiol (2ME2) is a natural metabolite of estradiol with recognized antiangiogenic and antitumor properties. These two properties are also shared by antiestrogenic compounds belonging to either selective estrogen receptor disruptor (SERD) or selective estrogen receptor modulator (SERM) types. 2ME2 as well as SERMs and SERDs have been shown potent inducers of apoptosis in MM cells both *in vitro *and *in vivo*. This brief review focuses on preclinical studies of 2ME2, SERD and SERM actions and discusses the benefit of such compounds in a therapeutic perspective.

### Effects of 2ME2 in MM

**Figure 1:**
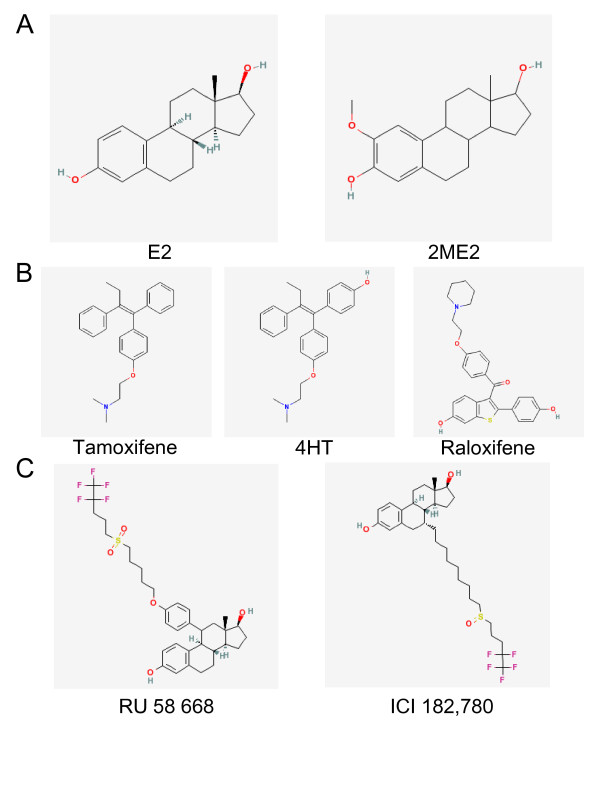
**Chemical structures of estrogenic and antiestrogenic molecules.** Chemical structures were obtained from PubChem Compound 47.

2ME2 is a natural metabolite of estradiol (Figure 1A) which possesses antitumoral and antiangiogenic activities on a wide spectrum of solid tumors and leukemias [3]. 2ME2 inhibits cell proliferation and induces apoptosis of MM cell lines, MM primary cells and engrafted tumors in immunodeficient mice [4,5]. *In vitro*, 2ME2, at micromolar concentrations (10–50 μM), has a selective activity on malignant MM cells since it displays no effects on normal B lymphocytes [4]. 2ME2 induces a G2-M phase arrest and triggers a mitochondrial-dependent cell death through the cytosolic release of cytochrome c and Smac and in turn, the activation of caspase-9 and thereafter, the activation of the executioner caspase-3 [4]. *In vivo*, 2ME2 or 2ME2-loaded liposomes affect xenograft tumors growth [4,5] and 2ME2 reduces significantly intratumoral microvessel density [4]. Microarray analyses identified genes modulated by 2ME2 and among them, genes regulating cell death/repair machineries, genes involved in the unfolded protein response or in the endoplasmic reticulum stress response, genes regulating proliferation/adhesion pathways and structural genes [6]. The same study demonstrated also that 2ME2 down-regulates c-Myc and targets p27^Kip1 ^which is cleaved to achieve its effects.

### Effects of estradiol in MM

The effects of 17-β-estradiol (E2, Figure 1A) on MM cells are less clear and data from the literature are more or less controversial. It has been shown that E2, also at micromolar concentrations, abolishes interleukin (IL)-6-dependent MM proliferation, an effect which is reversed by the estrogen receptor (ER) pure antagonist: ICI 182,780 (ICI). Indeed, E2/ER complexes induce the expression of PIAS3 (protein inhibitor of activated STAT3), one inhibitor of activated STAT3 (signal transducer and activator of transcription 3) at the transcriptional level [7]. IL-6, which plays a major role in the physiopathology of MM, regulates both cell survival and proliferation through the STAT3 pathway which is often constitutively activated in MM cells [2]. Inhibition of the STAT3 pathway induces MM cell apoptosis *in vitro *[8,9]. In that sense, STAT3 can be envisaged as a prime target for therapeutic intervention. Otsuki, his coworkers and us noted an inhibition of cell proliferation of most (but not all) MM cell lines in the presence of E2 [10,11] and a further inhibition of proliferation after AE treatment in the presence of E2 [10]. By contrast, the data of Treon and colleagues do not support such a role. Indeed, in their study, E2-treatment has no effect on MM cell lines [12]. The results of the different teams are reported in Table 1. It appears that the response to E2 could be cell-specific. Two interpretations are possible: a) in MM cells, the response to E2 is ER-dependent and some cells such as Karpas 620, OPM-2 or ARH-77 do not express functional ER and/or associated transcription cofactors; b) the response to E2 is ER-independent and E2-resistant cells lack signalization molecules engaged in the survival, proliferation or apoptosis pathways necessary for growth inhibition. Two cell lines U266 and RPMI 8226 behave differently according to the study; both are ancient, established cell lines which could have derived in cultures and could be no longer identical within the various places. Moreover, the techniques used for establishing inhibition of proliferation are quite different in the various studies (Table 1).

**Table 1:** Effects of E2 on proliferation of MM cell lines*

MM cell line	Response	Reference
ANBL6	Inhibition	[7]
ARH-77	No effect	[12]
KAS-6/1	Inhibition	[7]
KMM-1	Inhibition	[10]
LP-1	Inhibition	[11]
MM.1S/R	No effect	[4]
NCI-H929	Inhibition	[11]
OCI-My5	No effect	[12]
OPM-2	No effect	[11]
RPMI 8226	No effect	[4]
	Inhibition	[10]
	Inhibition	[11]
	No effect	[12]
U266	Inhibition	[10]
	Inhibition	[11]
	No effect	[12]

* Are presented in this table only authenticated MM (or at least well-characterized) cell lines indexed in [49]. In all cases, MM cell lines were treated *in vitro *with micromolar concentrations of E2 varying from 0.5 μM to 50 μM; various techniques were used to quantify cell proliferation: [^3^H]-thymidine incorporation, MTS reduction assay, cell number counting after trypan blue exclusion.

### Activities of SERMs in MM

SERMs comprise triphenylethylene compounds such as tamoxifene (Nolvadex) or toremifene (Fareston), benzothiophen derivatives such as raloxifene (Evista) and a small group of benzopyran derivatives (Figure 1B). They are therapeutic agents used for the prevention and the treatment of diseases such as osteoporosis (raloxifene) and breast cancers (tamoxifene, toremifene) [13]. In MM cells, they display a potent antiproliferative effect. Tamoxifene and its active metabolite 4-hydroxytamoxifene (4HT), inhibit MM cell proliferation [10,12,14] by blocking cells at the G1 phase [11] and by inducing apoptosis [11,12]. MM cell lines and primary cells are sensitive to this treatment. Importantly, the apoptosis of cells isolated from MM patients is obtained with concentrations of tamoxifene which do not alter the *in vitro *differentiation of hematopoietic progenitors into myeloid and erythroid lineages [12]. Toremifene exhibits the same biological activity although it seems less potent [10,11]. Raloxifene has also an antimyeloma activity through an arrest of the cell cycle at the S or G2/M phases depending on the cell line tested and the induction of a caspase-9/-8-dependent apoptosis [14]. Interestingly, microarray analyses showed that raloxifene treatment decreases the expression of genes involved in cell survival (including c-Myc) and induces the expression of genes regulating cell cycle [14]. In good agreement with that, we have reported that the effects of 4HT are mediated by a rapid (2–6 h post-treatment) down-regulation of c-Myc [11]. The effects of SERMs in xenograft models have not been reported so far.

### Activities of SERDs in MM

SERDs are also called pure antiestrogens since they have antiestrogenic effects in a majority of tissues even in the absence of estradiol. Most of them possess a steroidal backbone and act as estrogen inhibitors (Figure 1C). ICI, known as Faslodex, is used in the treatment of advanced breast cancers with tamoxifene-acquired resistance [15]. ICI induces an inhibition of MM cell proliferation although smaller than that induced by SERMs [12] (Gauduchon et al, submitted). RU 58 886 (RU) also a promising therapeutic agent for breast cancers [15] has the same antimyeloma activity (Gauduchon et al, submitted). RU-mediated inhibition of proliferation occurs through two independent processes: cell cycle arrest at the G1 phase and induction of mitochondrial- and endoplasmic reticulum-dependent apoptosis. We have found that c-Myc is the primary target of RU in MM cells and that the completion of apoptosis necessitates the cleavage of p27^Kip1 ^(Gauduchon et al, submitted). *In vivo*, RU-loaded liposomes impair engrafted tumor cells growth. In fact, an enhanced apoptotic process occurs in RU-treated animals, an apoptosis mediated by the mitochondrial intrinsic death pathway. Besides proapoptotic properties, RU is also capable of reducing tumor vasculature [16]. Indeed, in mice bearing RPMI 8226 tumors, stealth RU-loaded liposomes weekly injected *i.v*. accumulate at the vicinity of the microvessels surrounding the tumor. A high RU concentration is obtained locally due to AE release from the liposomes. After endocytosis of the liposomes or passive diffusion, RU has two effects within the cells: it blocks the production of VEGF (vascular endothelial growth factor) by MM cells and inhibits VEGF secretion by endothelial cells [16]. Those combined effects of encapsulated SERM and SERD on both tumoral cells and tumoral environment have been previously described in a xenograft model of breast cancer tumor [17].

## Are ER needed for MM response towards estrogenic or antiestrogenic compounds?

As summarized Table 2, 2ME2, SERDs and SERMs seem very potent in MM cell lines, inducing cell cycle arrest and/or apoptosis both leading to the inhibition of proliferation. The biological effects of estrogens are principally mediated by two types of receptors, namely ERα and ERβ which possess similar structures but distinct functions [18]. MM cell lines and primary cells express ERα and ERβ mRNAs [10] and proteins although with various levels [7,10,11,14,19,20] (Table 3). The relative level of each form remains unknown essentially because of the poor quality of most available antibodies and the variability of such antibodies among the studies. This point is very important to resolve since ERβ could be a physiological dominant negative form of ERα and the ERα/ERβ ratio may regulate the response to E2. Indeed, it has been reported that in mature B cells, and in B tumoral cells including LP-1 cell line, ERβ is abundant whereas ERα is not detectable [20]. MM cells may express predominantly ERβ form and be growth-inhibited by E2. A comparative analysis of the response of cells to E2 and the expression of ER types does not clarify further this point (see Tables 1 and 3). Interestingly, at high concentration (~10 μM), E2 inhibits cell proliferation and induces apoptosis of breast cancer cells suggesting a common mechanism of action [21]. Furthermore, ERβ inhibits angiogenesis and growth of breast cancer xenografts [22]. The emerging hypothesis is that, in MM cells, E2 may signal through ERβ to inhibit cell proliferation *in vitro *and *in vivo*.

**Table 2:** Response of MM cell lines towards various treatments

Cell lines	Compounds							**Ref**.
	**2ME2**	**Tam**	**4HT**	**Tor**	**Ral**	**ICI**	**RU**	

ANBL6	CCA + Ap.*							[5]
Karpas 620			No					[11]
							No	**

KAS-6/1	CCA + Ap.							[5]
KMM-1		CCA + Ap.		CCA + Ap.				[10]
LP-1			CCA + Ap.					[11]
						CCA	CCA	**

MM.1S/R	Ap.							[4]
NCI-H929			CCA + Ap.					[11]
						Ap.	Ap.	**
OCI-My5	CCA + Ap.							[4]
		CCA + Ap.						[5]

OPM-2			No					[11]
						CCA	CCA	**
RPMI 8226	Ap.							[4]
	CCA							[5]
			CCA + Ap.					[11]
	CCA + Ap.		CCA + Ap.	No		CCA + Ap.		[12]
		CCA + Ap.			CCA + Ap.			[13]
						Ap.	Ap.	**
U266		CCA + Ap.		CCA + Ap.				[10]
			CCA + Ap.					[11]
		CCA + Ap.	CCA + Ap.			CCA + Ap.		[12]
		CCA + Ap.			CCA + Ap.			[14]
						No	No	**

Presented cell lines were chosen as in Table 1. Cell lines were treated *in vitro *with micromolar concentrations of 2ME2, SERMs or SERDs (0.5–50 μM) and drug effects were detected 24–72 h later. Cell cycle arrest was demonstrated essentially by flow cytometry sorting of propidium iodide-stained cells, apoptosis was assessed with different techniques such as annexin V staining, caspase activity assays, mitochondrial membrane potential measurement, and TUNEL method. * CCA, cell cycle arrest; Ap., apoptosis; Tam, tamoxifene; Tor, toremifene, Ral, raloxifene. ** Gauduchon et al, submitted.

**Table 3:** Expression of ERα and ERβ on MM cell lines

Cell line	ERα	Antibody	ERβ	Antibody	Reference
ANBL6	+++	nd, Upstate	-	nd, Upstate	[7]
Karpas 620	-	D12, Santa Cruz	-	G Greene, E82	[19]
			-	J-C Faye	[11]
KAS-6/1	+++	nd, Upstate	+	nd, Upstate	[7]
LP-1	-	D12, Santa Cruz	+	J-C Faye	[11]
	-	D12, Santa Cruz	+	G Greene, E82	[19]
	-	HC-20, Santa Cruz	+++	503	[18]
	+	1D5, Dako			[18]
NCI-H929	+++	D12, Santa Cruz	+	J-C Faye	[11]
OCI-My5	-	TE1115011			[12]
OPM-2	+	D12, Santa Cruz	+	J-C Faye	[11]
RPMI 8226	+	D12, Santa Cruz	+	J-C Faye	[11]
	+++	HC-20, Santa Cruz	+++	H-150, Santa Cruz	[14]
	+	TE1115011			[12]
U266	+++	HC-20, Santa Cruz	+	G Greene, E82	[19]
			+	J-C Faye	[11]
	+++	TE1115011, nd			[12]

Presented cell lines were chosen as in Table 1. Proteins were purified from cultured MM cell lines, separated by SDS-PAGE and immunoblotted with the indicated antibodies according to conventional methods. +++, high expression; +, weak expression; -, no expression; nd, not described.

The necessity of ER for the antiproliferative response to 2ME2 has been ruled out in breast cancer cells [21]. 2ME2 binds poorly to ERs, is not agonistic for ERs and its antiproliferative activity is mediated independently of both forms. These findings coupled to other studies suggest that 2ME2-mediated effects on MM cells are independent of ERs [4].

The question of ERs mediating the AE response in MM cells is still open. Karpas 620 cells, which do not express any ER isoforms, are completely resistant to 4HT [11]. When transfected with expression plasmids coding for ERα or ERβ, and then treated with 4HT, Karpas 620 cells exhibit a weak but significant decrease of cell proliferation [11]. This result indicates that ERα and ERβ restaure at least partially the response to 4HT. OPM-2 cells although expressing moderately both ERα and ERβ, appear sensitive to ICI and RU but resistant to 4HT. The opposite is also true, U266 highly expressing ERα, is resistant to ICI and RU but 4HT-responsive [11] (Gauduchon et al, submitted). OCI-My5 cells which are ERα-negative are growth-inhibited after 2ME2 [4] and Tam [5] treatments. Altogether, these results indicate that ERα or ERβ may contribute but only partially to the response against AEs. Moreover, the presence or the absence of a specific ER form cannot be correlated to a particular response to estrogenic or antiestrogenic compounds (see Tables 2 and 3).

The effects of SERMs and SERDs on breast cancer cells resemble the effects on MM cells. Indeed, *in vitro *concentrations of SERMs and SERDs necessary to achieve the biological response are close to 10 μM [23,24]. Moreover, ICI and tamoxifene, have been shown to induce a rapid cell death in MDA-MB-231 ER-negative breast cancer cells, like in ER-positive MCF-7 cells [25]. Tamoxifene- or ICI-induced breast cancer cells apoptosis is associated with the increase of tumor necrosis factor receptor 1 (TNFR1) [26], the activation of caspase-3 and c-jun NH2-terminal kinase-1 (JNK) [25] or the extracellular signal-regulated kinase (ERK) [27] signaling pathways, or an increase in the production of oxygen reactive species [28]. Tamoxifen and ICI, although being strong affinity ligands for ERs, act on the viability of ER-positive and -negative cells *via *multiple signaling pathways. The same multiple signaling pathways could be implicated in MM cells.

E2 as well as AEs may induce a cellular response through non genomic signals mediated by membrane-associated ERs [29]. Several works have demonstrated the membrane localization of ERα in MCF-7 cells and other cell types [30-32]. Such localization may explain the rapid effect of E2 and AE on ERK and their subsequent actions [27]. Today, no data support a membrane localization of ERβ. Interestingly also, a raft-located estrogen receptor-like protein, distinct from ERα, insensitive to ICI, has been characterized [33]. Thus, it is possible that the effects of estrogen in many cells and that of AEs in MM cells are mediated by a still unknown receptor.

## How 2ME2, SERMs and SERDs signal to arrest the cell cycle and to trigger apoptosis?

**Figure 2:**
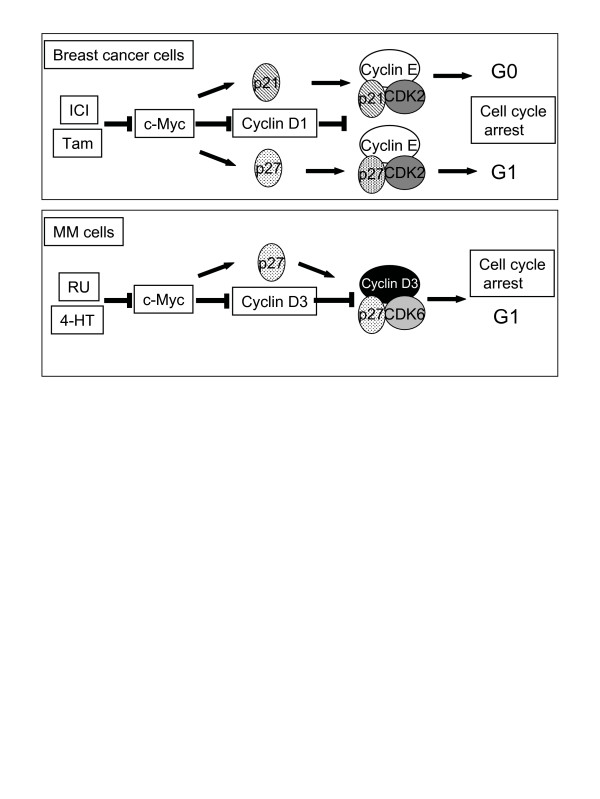
**Schematic representation of AEs signaling in breast cancer and myeloma cells. **In breast cancer cells, ICI and tamoxifene (Tam) both downregulate c-Myc and its target cyclin D1 [35, 39]. But depending on the treatment, there is either an upregulation of p21^Cip1 ^or p27^Kip1 ^and thereafter the inhibition of cyclin E/CDK2 activity and the arrest in G0 for ICI or G1 for Tam [36]. In MM cells, 4HT and RU treatments induce a rapid downregulation of c-Myc, an upregulation of p27^Kip1 ^and the subsequent decrease activity of cyclin D/CDK leading to a G1-arrest in [11] (Gauduchon et al, submitted).

**Figure 3:**
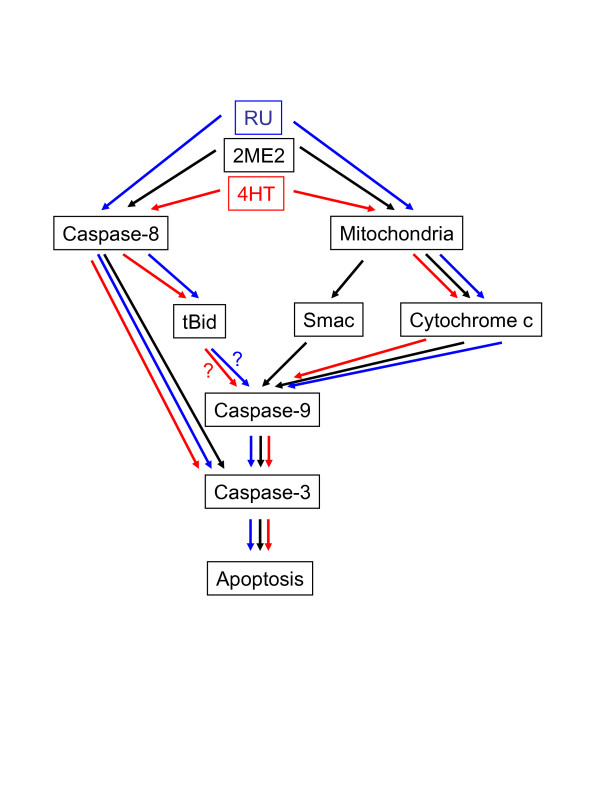
**Schematic representation of apoptotic pathways triggered by 2ME2, 4HT and RU.** Apoptosis induced by 2ME2 (in black) is mediated by the release of apoptogenic proteins cytochrome c and Smac from the mitochondria to the cytosol, followed by the activation of caspase-9, -8 and -3 [4]. The mitochondria intrinsic death pathway is also recruited by 4HT (in red) and RU (in blue): following the cytosolic release of cytochrome c, the caspase-9 is activated then the caspase-3 [11, 16] (Gauduchon et al, submitted). 4HT and RU treatments also lead to the activation of caspase-8 but we have not yet determined the mechanism of caspase-8 activation. However, we know that the three death receptors selectively expressed by MM cells, Fas, DR4 and DR5 [48] are not involved (data not shown).

2ME2 and SERMs/SERDs compounds inhibit MM cell proliferation mainly by two distinct and independent ways: they arrest cell cycle and they induce apoptosis. It appears that all these compounds although structurally, biologically different signal through the same pathways. The first common target of 2ME2, raloxifene, 4HT and RU is the proto-oncogene c-Myc which is involved in the control of proliferation [6,11,14] (Gauduchon et al, submitted). c-Myc control the expression of G1 transition genes and inhibiting c-Myc halts tumor cell proliferation [34]. This is the precisely the biological activity of ICI and tamoxifen in breast cancer cells, they downregulate c-Myc and induce cell cycle arrest through the induction of the two cell cycle inhibitors p21^Cip1 ^or p27^Kip1 ^(Figure 2) [35-38]. Following the downregulation of c-Myc in 4HT- or raloxifene-treated MM cells, we and others observed the up-regulation and redistribution of p21^Cip1 ^and p27^Kip1 ^leading to cell cycle arrest [11,14]. Interestingly, p27^Kip1 ^is degraded and cleaved by a protease in 2ME2-induced apoptotic MM cells [6] that we identified as a caspase in RU-treated cells (Gauduchon et al, submitted). The apoptosis is triggered by the mitochondrial intrinsic death pathway by AE (Figure 3) again in close similarity with that observed in breast cancer cells [39]. Importantly, also, SERMs could interfere with survival pathways known to be constitutively activated in MM such as Janus Kinase (JaK)/STAT3 [40,41], NF-κB (nuclear factor-κB) [42,43] and Ras/MAPK (mitogen-activated protein kinase) [44,45]. For example, raloxifene blocks the NF-κB activity through a modulation of the ER association with the p65 subunit [14]. We have some preliminary data which indicate that RU is capable of inhibiting the JAK/STAT3 but not the Pi3K (phosphoinositol 3-kinase)/AKt pathway (Seguin et al, unpublished).

However, we did not find any cross-talk between ER and STAT3 in RU-treated MM cells. In fact, we think that in the cellular model used by Wang and coworkers, the STAT3 pathway is functional [7]. This is not true for the MM cell lines used in our study (they all display a constitutively activated STAT3) and for MM patients in which STAT3 is also constitutively activated [40,41].

## Conclusion

To conclude, 2ME2 and SERMs (which seem more efficient than SERDs) are able to block MM cell cycle arrest, to trigger the intrinsic death pathway, to inhibit one or several survival pathways. These properties are necessary both to limit MM cell proliferation and to overcome resistance towards conventional or more novel drugs. Indeed, most of these compounds are able to synergize with dexamethasone, doxorubicin, bortezomib, arsenic trioxide to achieve cell growth inhibition [5,11,13] (Gauduchon et al, submitted). Moreover they are well-tolerated; and encouraging preliminary results have been reported on MM patients receiving tamoxifene [46]. Finally, 2ME2, SERMs/SERDs target the tumoral microenvironment and in particular inhibit the synthesis of angiogenic factor(s); all these properties provide a rationale for clinical studies.

## Competing interests

The author(s) declare that they have no competing interests.

## Authors' contributions

BS and JMR both contributed to the conception of the paper, drafted the manuscript, read and approved the final version.
